# Editorial: Psychobiological Research in Psychosomatic Medicine

**DOI:** 10.3389/fpsyt.2017.00019

**Published:** 2017-02-13

**Authors:** Silvia Ouakinin, Marco Torrado, Susana Eusébio, David Barreira

**Affiliations:** ^1^Faculty of Medicine, Department of Psychiatry and Medical Psychology, University of Lisbon, Lisbon, Portugal

**Keywords:** psychosomatic, mind–body interactions, psychophysiology, chronic medical conditions, psychobiological research

**Editorial on the Research Topic**

**Psychobiological Research in Psychosomatic Medicine**

In the launching of “Psychobiological Research in Psychosomatic Medicine” topic, we defined the principal goal of psychosomatic medicine as “the integration of biological, psychological, and social systems that may influence health or pathology, namely in chronic diseases and co-morbid physical and psychiatric disorders.” According to Fava et al. (1) in the interdisciplinary field of Psychosomatic Medicine, psychosocial factors influencing individual vulnerability to diseases include life events and allostatic load, health attitudes and behaviors, social support, psychological well-being, spirituality, and personality. The Diagnostic Criteria for Psychosomatic Research facilitated the translation of psychosocial variables that derived from psychosomatic research into operational tools, imperative in a clinical setting. From neurosciences perspective the investigation should clarify neural processes that may link psychosocial stressors and inflammation, in several pathologies, such as cancer (2).

In the present topic, we attempted to promote a scientific forum for exploring these multiple pathways. Indeed, psychobiological research, in our times, can go through a wide range of levels. Departing from molecular analysis, we can hope to be able to infer a sort of biological signatures associated with particular symptom clusters, in a dimensional approach, or with different diseases, as discrete entities. From a psychophysiological level, we may pursue the comprehension of the pathways that link the effects of early childhood adversity, chronic stress, social support and health, through neuroendocrine and autonomic mechanisms that determine the stress responses. At a macroscopic level, we can explore the role of individual sociodemographic variables such as ethnicity, personality, treatment modalities, and health promotion through psycho-educational interventions.

The collected papers present different contributions for understanding the variability of processes that make more evident the link between individual differences (at organic and psychosocial levels) and illness.

Coelho et al., using animal models, explored the role of adenosine, which acts as a neuromodulator in several brain areas and seems to play an important influence on other neurotransmitters, also implicated in a wide range of brain processes and diseases. They studied the interaction of A_2A_R receptors and dopamine, particularly the impact of A_2A_R overexpression in cortical areas in dopamine related behaviors. This overexpression in hippocampus, cortex, and striatum is associated with depressive-like behaviors and increased locomotor activity. Additionally, the A_2A_R overexpression in forebrain is related to depression, which may explain the depressive signs seen in aging, chronic stress, and Alzheimer’s disease.

Perhaps in a psychophysiological perspective, we achieved a better understanding of the relations between mental and physical states, but specificity is lacking. As Chalmers et al. described in their meta-analysis, anxiety disorders are associated with lower heart rate variability, a well-known marker of cardiovascular risk. However, the specificity of different affective states is not easy to capture according to the state of the art. Despite the clinical evidence of psychosomatic mechanisms in health and diseases, the control of several confounders that probably prevent a more comprehensive integration of data is needed.

In fact, the paper by Assari and Lankarani, supporting the differences in the association between negative affect and chronical medical conditions in black and white Americans, in the framework of the so called “Black-White Health Paradox,” need to be contextualized in socio-cultural and economic determinants of health. Also social support, both in instrumental and affective dimensions, is widely recognized as a moderator of the impact of negative events and their correlated affective states, in psychophysiological balance.

Papers focused on potential interference of psychological factors shed light on the importance of personality variables in health behaviors and their impact in disease expression and progression. In this sense, Conti et al. provided a systematic review on the links between Type D personality and diabetes. Through a total of seven research studies, the authors concluded that Type D personality seems to negatively interfere with clinical features in diabetes. Specifically, it seems to predict both poor therapeutic adherence and unhealthy behaviors due to increased distress (i.e., depressed mood, anhedonia, and anxiety). Considering the high prevalence of Type D personality among diabetes patients, the authors highlight the clinical relevance of an early personality assessment, to prevent medical complications due to poor adherence.

Considering treatment modalities as a level of mind–body integration, current research allows an improvement in the ability to relieve symptoms, even though the etiological mechanisms remain not clear. Bechter et al. present a clinical case of tinnitus remission after cervical collar use. Following symptom remission, tinnitus was again induced by variations of head inclination, which led authors to hypothesize a possible pathogenic relationship of muscle tension of the upper posterior cranial transition and tinnitus. Even with limitations, their findings point to a complex myriad of factors, including psychological ones, which may trigger the onset or the maintenance of the symptoms. Nevertheless, evidence supporting these potential pathological mechanisms is still lacking.

Keeping in mind the association between health outcomes and psychological factors in the context of chronic conditions, Ribeiro et al. have also pointed that psycho-educational interventions in HIV patients’ treatment can make a difference. In their longitudinal study, a psycho-educational program to promote Highly Active Antiretroviral Therapy (HAART) adherence was implemented for patients in two groups, adherents, and non-adherents. The follow-up showed that the number of non-adherents decreased significantly after intervention. Better results in CD4 T lymphocytes and viral load were achieved, suggesting that this kind of interventions is cost-effective since they improve adherence to HAART.

This topic addresses the importance of an integrative multi-level approach, in which disease determinants may interact according to environmental, relational, individual, and biological levels, in a systemic model. Chronic diseases are the main field where the psychophysical relations are disclosed, being the integration of different evidences a major challenge for the therapeutic setting and delivery of care. Psychobiological research in psychosomatic medicine, complemented by the necessary reflexive and clinical dimensions can be conceptualized as an alternative response to the traditional dualistic attitude in medical sciences.

Regardless the boundaries of current knowledge in the mind–body paradigm, the collected papers brought new contributions for understanding some puzzling issues in a psychosomatic approach.

## Author Contributions

All authors wrote the editorial together and approved its final version.

## Conflict of Interest Statement

The authors declare that this work was conducted in the absence of any commercial or financial relationship that could be construed as a potential conflict of interest.
